# Genetic polymorphisms associated with adverse pregnancy outcomes in nulliparas

**DOI:** 10.1038/s41598-024-61218-9

**Published:** 2024-05-07

**Authors:** Raiyan R. Khan, Rafael F. Guerrero, Ronald J. Wapner, Matthew W. Hahn, Anita Raja, Ansaf Salleb-Aouissi, William A. Grobman, Hyagriv Simhan, Robert M. Silver, Judith H. Chung, Uma M. Reddy, Predrag Radivojac, Itsik Pe’er, David M. Haas

**Affiliations:** 1 Department of Computer Science, Columbia University, New York, NY USA; 2 Department of Biological Sciences, North Carolina State University, Raleigh, NC USA; 3 Department of Computer Science, Indiana University, Bloomington, IN USA; 4 Department of Biology, Indiana University, Bloomington, IN USA; 5 Department of Computer Science, CUNY Hunter College, New York, NY USA; 6 Department of Obstetrics and Gynecology, Northwestern University Feinberg School of Medicine, Chicago, IL USA; 7 University of Pittsburgh Medical Center, Pittsburgh, PA USA; 8 Department of Obstetrics and Gynecology, University of Utah, Salt Lake City, UT USA; 9 Department of Obstetrics and Gynecology, University of California, Irvine, Orange, CA USA; 10 Department of Obstetrics and Gynecology, Columbia University, New York, NY USA; 11 Khoury College of Computer Sciences, Northeastern University, Boston, MA USA; 12 Indiana University School of Medicine, Indianapolis, IN 46202 USA

**Keywords:** Genetic association, Preeclampsia, Preterm birth, Gestational diabetes, Fetal death, Stillbirth, Pregnancy loss, Miscarriage, Genome-wide association studies, Risk factors

## Abstract

Adverse pregnancy outcomes (APOs) affect a large proportion of pregnancies and represent an important cause of morbidity and mortality worldwide. Yet the pathophysiology of APOs is poorly understood, limiting our ability to prevent and treat these conditions. To search for genetic markers of maternal risk for four APOs, we performed multi-ancestry genome-wide association studies (GWAS) for pregnancy loss, gestational length, gestational diabetes, and preeclampsia. We clustered participants by their genetic ancestry and focused our analyses on three sub-cohorts with the largest sample sizes: European, African, and Admixed American. Association tests were carried out separately for each sub-cohort and then meta-analyzed together. Two novel loci were significantly associated with an increased risk of pregnancy loss: a cluster of SNPs located downstream of the *TRMU* gene (top SNP: rs142795512), and the SNP rs62021480 near *RGMA*. In the GWAS of gestational length we identified two new variants, rs2550487 and rs58548906 near *WFDC1* and *AC005052.1*, respectively. Lastly, three new loci were significantly associated with gestational diabetes (top SNPs: rs72956265, rs10890563, rs79596863), located on or near *ZBTB20*, *GUCY1A2*, and *RPL7P20*, respectively. Fourteen loci previously correlated with preterm birth, gestational diabetes, and preeclampsia were found to be associated with these outcomes as well.

## Introduction

Adverse pregnancy outcomes (APOs) are a serious threat to the health of pregnant persons and children. APOs affect a significant fraction of pregnancies across the globe and are among the leading causes of morbidity and mortality worldwide^1^. Among the most common APOs are preterm birth (which occurs in over 10% of pregnancies in the United States (US)^2^), preeclampsia (which develops in 5–10% of pregnancies^3^), gestational diabetes (occurring in roughly 6% of pregnancies in the US^4^), and pregnancy loss (estimated to occur in about 7–14% of pregnancies^5,6^). APOs are also highly correlated with future disease in birthing parents. For example, gestational diabetes carries a lifetime 50% risk of type 2 diabetes (T2D) in the mother^7^, while preeclampsia is associated with a 2–threefold increase of cardiovascular disease later in life^8^. Yet, factors driving these diseases remain poorly understood, hindering efforts in prevention and treatment.

To better understand the mechanisms and improve prediction of APOs in nulliparous individuals, the Nulliparous Pregnancy Outcomes Study: Monitoring Mothers-to-Be (nuMoM2b) consortium^9,10^ recruited and prospectively followed a large cohort of nulliparous people beginning in their first trimester of pregnancy. Participants underwent several assessments over the course of their pregnancies, resulting in a comprehensive profile that included biospecimens, clinical measurements, ultrasounds, behavior (through interviews and questionnaires), physical activity assessment, and dietary content.

By precisely characterizing different aspects of over 10,000 pregnancies, the nuMoM2b cohort has already yielded valuable insights into factors that contribute to APOs^11,12^. Additionally, the availability of biospecimens provides a unique opportunity to study the genetic underpinnings of APOs. The objective of this study was to test for association between common variants across the maternal genome and four APOs in the nuMoM2b cohort: gestational length (as a proxy for preterm birth), preeclampsia, gestational diabetes mellitus (GDM), and pregnancy loss. Our investigation leverages ancestrally diverse populations to further isolate potential genetic factors involved in these APOs.

## Methods

### Participants

The participants of the analysis were enrolled in the nuMoM2b cohort (https://www.nichd.nih.gov/research/supported/nuMoM2b), a longitudinal, multiethnic cohort study of nulliparous individuals. All participating centers, documented in Haas et al.^10^, obtained approval by the local Institutional Review Boards (IRBs) of their corresponding recruitment institutions. The genotype analysis is covered by Indiana University’s IRB, which was Protocol Study number 1008–08, approved on 9/28/2010. This study was conducted in accordance with the ethical principles of the Declaration of Helsinki. All participants included in this study provided informed consent. The study enrolled 10,038 nulliparous people from the first trimester of their pregnancy to participate in three study visits during pregnancy. Collection of health status and biomarkers were conducted at regular intervals, and documentation of pregnancy outcomes was performed by medical record abstraction using a priori definitions (details of this process were described by Haas et al.^9,10^).

### Phenotype definitions

Pregnancy loss: All subjects who had a pregnancy loss, regardless of gestational age, were considered as cases (Table 1). A pregnancy loss occurs when the fetus dies during gestation. This categorization includes all cases of fetal demise occurring under 20 weeks of gestation, and all stillbirths (defined as a fetal death occurring from 20 weeks of gestation onward). Individuals who underwent termination of pregnancy were excluded from the pregnancy loss analysis. Subjects who had a live birth were treated as controls.

**Table 1:** Number of subjects in the nuMoM2b cohort used for the GWAS.

	African Ancestry				Admixed American Ancestry				European Ancestry			
Initial	1425				846				6082			
Preprocessing	1384				811				5896			
After removal of related subjects	1374				811				5891			
GWAS	GDM	GL	PEC	PL	GDM	GL	PEC	PL	GDM	GL	PEC	PL
Available phenotypes	1258	1355	1355	1308	754	775	775	775	5605	5726	5779	5726
Live and spontaneous birth	–	770	–	–	–	512	–	–	–	3499	–	–

Each row represents the number of subjects remaining after applying the step. Bolded numbers represent the final number of subjects used for each separate GWAS (GDM: gestational diabetes, GL: gestational length, PEC: preeclampsia, PL: pregnancy loss).

Gestational length: We opted to use a quantitative phenotype, gestational length, instead of a binary preterm/full term outcome to gain additional information and statistical power by using a more granular phenotype. Gestational length was determined from an estimated due date established by a first-trimester ultrasound crown-rump length measurement and was recorded in weeks^10^. Preterm birth was defined as any live birth that occurred before 37 weeks gestational age. Cases of stillbirth, fetal demise, and termination (elective and indicated) were all excluded from this phenotype group (Table 1). Cases of preterm birth that were medically indicated (e.g. for preeclampsia), were excluded. Term births that had labor induced or were by planned cesarean delivery were included as they occurred at term and because gestational age at delivery is a right-bordered outcome. Thus, we did not believe that their inclusion would alter this result and excluding these cases would limit the sample size of deliveries > 37 weeks, potentially skewing the data toward lower gestational age means.

Gestational diabetes (GDM): GDM was diagnosed through clinical evaluation, from fasting blood sugar, sequential 1-h glucose challenge test followed by a 3-h glucose tolerance test (GTT), or a single step 2-h 75-g GTT^13^. We excluded individuals diagnosed with pregestational diabetes from GDM analyses, and all other individuals were treated as controls.

Preeclampsia: Cases are individuals with a diagnosis of preeclampsia (with and without severe features), eclampsia, and chronic hypertension with superimposed preeclampsia. A detailed description of nuMoM2b study definitions of hypertensive disorders of pregnancy was published in the supplement to the paper by Facco et al.^13^. All other individuals were treated as controls. We did not exclude individuals with hypertension antepartum, as we are interested in unearthing markers specific to gestational disease, not general hypertension.

### Genotyping

We genotyped all participants who had adequate samples and agreed to be genotyped (n = 9,757). We used the Infinium Multi-Ethnic Global Array (MEGA; Illumina, USA), which is designed to adequately query individuals of multiple genetic ancestries^14^ (a known issue in genotyping studies), enriched for variants of clinical importance^15^, and has been successfully used to study recent admixed populations^14,16^. The MEGA allowed us to genotype > 1 million variants that are on average 1.4 Kb apart, effectively covering the entire genome. DNA extractions from whole blood were carried out on a QIAsymphony instrument (from Qiagen; extraction kit DSP DNA Midi Kit #937,355, protocol Blood_1000_V7_DSP) at the Center for Genomics and Bioinformatics (Indiana University, Bloomington), and genotyping was completed at the Van Andel Institute (Grand Rapids, MI, USA). We imposed standard quality control filters at this stage, all involving technical measurements of the raw intensity data (cluster separation < 0.3, normalized R-value mean < 0.2 for all genotypes, 10th percentile of the GenCall scores < 0.3) using GenomeStudio v2.4 (Illumina). Genotype calls for the ~ 1.7 million loci that passed initial quality control (98.3% of all markers in the array) were made with Beeline autoconvert (Ilumina).

### Quality control pipeline

**Figure 1:**
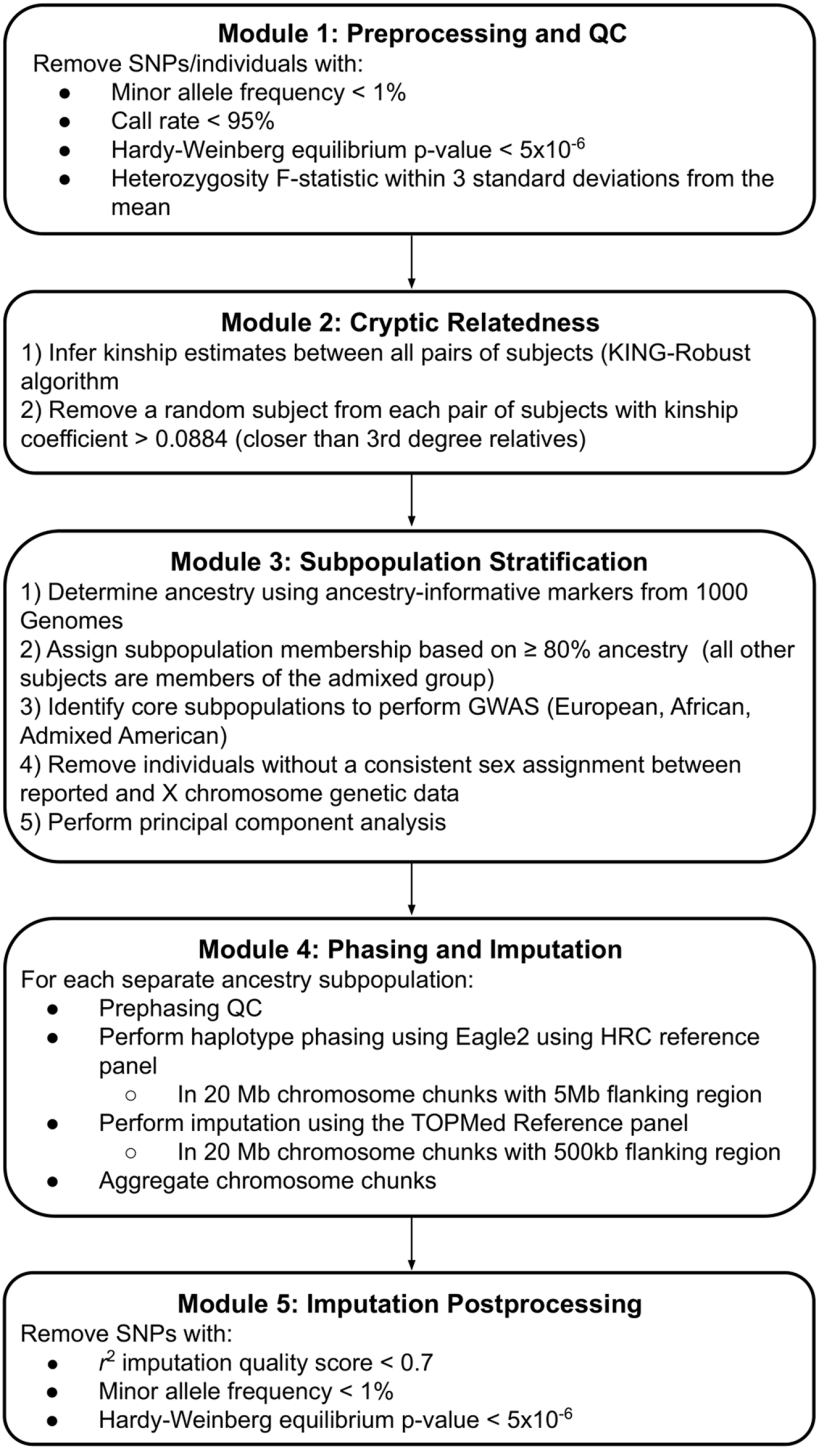
Overview of the quality control (QC) pipeline used to preprocess nuMoM2b subjects.

We leveraged a multi-step quality control (QC) pipeline, depicted in Fig. 1, to adequately address the heterogeneous nature of the dataset. The pipeline is broken into five separate modules that integrate current best practices in GWAS QC, which are further described in the text below. Unless otherwise stated, both the quality control steps and analyses were carried out using PLINK1.9^17^.

### Module 1: preprocessing and QC

The initial preprocessing of the dataset removed poorly genotyped individuals and SNPs according to the following criteria: (1) minor allele frequency (MAF) < 1%, (2) missingness of genotyping per individual and per marker > 5%, (3) Hardy–Weinberg Equilibrium (HWE) test p-value < 5 × 10^–6^, and (4) heterozygosity F-statistic within 3 standard deviations from the mean heterozygosity across all subjects using autosomes (Supplementary Figure S1). Significantly reduced heterozygosity may be indicative of high levels of consanguinity and subjects with excessive heterozygosity are suggestive of sample contamination, thus we excluded these subjects from the downstream dataset.

### Module 2: cryptic relatedness

The self-reported racial and ethnic diversity of the nuMoM2b cohort necessitates a careful approach in assessing relatedness. As the pairwise identity-by-descent (IBD) estimation implemented in PLINK assumes a homogeneous subset, we used the KING-Robust algorithm, a pairwise kinship estimator for GWAS that is robust to the presence of unknown population substructure^18^. We inferred kinship estimates between all pairs of subjects in the cohort (Supplementary Figure S2), randomly removing one subject from the pairs of subjects with first- or second-degree relatedness (kinship coefficient > 0.0884) such that we minimized the number of subjects removed.

### Module 3: subpopulation stratification

With the goal of minimizing spurious genetic associations in downstream analyses driven by population stratification and ancestry-based allele frequency differences, we clustered the cohort by genetic ancestry (Supplementary Figure S3). We determined the ancestry of each subject using SNPweights v.2.1 and a set of approximately 40,000 ancestry-informative markers curated by the 1000 Genomes Consortium^19^. Samples were clustered into five ancestry groups concordant with the 1000 Genomes Consortium^19^ subpopulations: African (AFR, n = 1425), Admixed American (AMR; n = 846), East Asian (EAS; n = 323), European (EUR, n = 6082), and South Asian (SAS; n = 112). Membership in each ancestry subpopulation was assigned based on having ≥ 80% ancestry in the specific subpopulation (Supplementary Figure S4). We observed a large fraction of highly admixed individuals; thus, we also established a sixth group (ADM, n = 891) of subjects who have no percent ancestry > 50% in any single ancestry group. Not all ancestry groups contained enough subjects to power a genome-wide association. Accordingly, we only proceeded with imputing the European, African, and Admixed American ancestry sub-cohorts. From this module onward, all QC steps are performed at the sub-cohort level.

Next, we checked the consistency of reported sex with sex assignments imputed from X chromosome breeding coefficients. This step was performed after subjects were grouped into subpopulations because F-statistic approximation for the X-chromosome relies on accurate MAF estimates, which vary at the subpopulation level. The sex check consists of four steps: (1) unambiguously re-coding the pseudo-autosomal region of the X-chromosome, (2) performing LD-based pruning on the set of markers used for the F-estimate, (3) confirming that all F estimates yield female calls using the threshold F < 0.6 (PLINK suggests a cutoff of F > 0.8 for a male call), and (4) removing any subjects with discordant results. Lastly, population structure was determined in PLINK using pruned SNPs from the data (linkage disequilibrium pruning *r*^2^ < 0.1). The top ten principal components were computed for each of the three subpopulations (EUR, AFR, AMR).

### Module 4: phasing and imputation

Subjects in these sub-cohorts were phased with Eagle2 and imputed by Minimac3^20^ using the TOPMED Imputation Server^21,22^. Prior to phasing, TOPMED partitions the data into 20 megabase length chunks and removes SNPs that are: (1) duplicates, (2) indels, (3) strand ambiguous (C/G and A/T), (4) not included in the Haplotype Reference Consortium (HRC) panel, and (5) mismatched between the reference panel and study. Data were then imputed using version R2 of the TOPMED panel, currently the largest panel of sequenced human genomes, and containing representation from the ancestry groups observed in nuMoM2b.

### Module 5: imputation postprocessing

Following imputation, we excluded SNPs with an *r*^2^ quality score < 0.7, MAF < 1%, and a Hardy–Weinberg Equilibrium (HWE) *P* < 5 × 10^–6^ within each imputed group.

### Genome-wide associations

Association testing was carried out using regression models implemented in PLINK v1.9^17^. The model was adjusted for each subject’s rank-transformed age, body mass index (BMI), and the first ten principal components from population structure analysis. As maternal age has a nonlinear effect on pregnancy^23,24^, we transformed each subject’s age into the distance from the median percentile in all subjects. The suggestive association threshold was *P* < 1 × 10^–5^ and the threshold for genome-wide significance was *P* < 5 × 10^–8^. Any subjects with missing age or phenotype information were excluded from analysis. The multi-ancestry results from the sub-cohorts were fixed-effect meta-analyzed using GWAMA^25^ and result plots were displayed using R libraries. As the majority of the subjects in nuMoM2b are of European ancestry, we only considered variants shared by this group, as the African and Admixed American sub-cohorts were underpowered in detecting signal in a standalone GWAS. Due to the smaller cohort size and large class imbalance (7 cases, 768 controls) observed in the Admixed American cohort for pregnancy loss outcomes, we did not perform a pregnancy loss GWAS for this sub-cohort.

Using the results of the genome-wide associations for each APO, we inferred SNP-based heritability of these traits using LDscore^26^. We scanned for putative regulatory effects by carrying out a transcriptome-wide association study (TWAS) as implemented in the Fusion software, and using the available expression reference weights for whole blood and adipose tissue^27^, as well as liver, pancreas, vagina, and uterus, and whole blood from the GTEx v7 multi-tissue RNA-seq data set. For TWAS, *P*-values were Bonferroni-corrected by the number of genes in each panel. Conditional expression analyses were performed using R scripts from the Fusion software. As both SNP-based heritability and TWAS inferences rely on reference panels, and relevant panels for these were developed with EUR cohorts, we used only EUR individuals in these two analyses.

### Variant fine mapping and annotation

We utilized the previously defined fine-mapping approach^28^ to delineate the 95% credible set for each significant meta-analysis locus containing more than one SNP of interest. In this approach, the posterior probability that each variant is causal is computed using approximations of the necessary Bayes factors. Only variants with a cumulative posterior probability above 95% are included in the credible set.

In characterizing the putative causal role of our variants of interest, we further annotated each SNP using Variant Effect Predictor^29^ (VEP) to identify the corresponding gene function, and RegulomeDB^30^ to understand the regulatory context. VEP enables genome interpretation by providing the gene and transcript level context, while also describing the regulatory location within the gene and type of point mutation for each variant. RegulomeDB heuristically scores variants on a scale of 1a (high confidence) to 7 (low confidence) given the presence of regulatory function as assessed by various functional screens, including annotations from ENCODE^31^ and other sources.

## Results

### Pregnancy loss

The multi-ancestry meta-analysis revealed a set of 12 novel SNP associations across two loci associated with pregnancy loss (Table 2 and Supplementary Table S1). Significant SNPs were located within or near the genes *TRMU* and *RGMA* (Table S1, Supplementary Figures S10-S12). While most of the associated SNPs appeared in both the European and African cohorts, two SNPs (rs143149726 and rs142372194) were only found in the European cohort (Supplementary Table S2). Additionally, fine-mapping the eleven SNPs identified on or near *TRMU* eliminated rs147382049 from the 95% credible set for the locus. For the nine remaining multi-ancestry variants, the direction of effect was concordant across the two ancestry cohorts and suggestive of an increased odds of pregnancy loss in carriers of the alternative allele. The genomic inflation factor was suggestive of minimal confounding effects in the meta-analysis (Supplementary Figure S5). This trait showed the highest SNP-based heritability (*h*^2^ = 0.30, SE = 0.075) of the four APOs studied.

**Table 2:** The top genome-wide SNPs across the three genome-wide associations with significant results.

APO	SNP	rsID	Beta/OR	*P*_*meta*_	Effect Direction	*P*_AFR_	*P*_EUR_	*P*_AMR_	Nearest gene
Gestational length	chr16:84,329,456:A:C	rs2550487	− 1.28	3.6 × 10^–9^	EUR (–)	N/A	2.86 × 10^–9^	N/A	WFDC1
	chrX:119,710,269:G:A	rs58548906	− 1.43	4.12 × 10^–8^	EUR (–) AFR (-) AMR (-)	0.00971	1.29 × 10^–12^	0.00732	AC005052.1
GDM	chr3:114,815,630:A:G	rs72956265	3.26	3.01 × 10^–8^	EUR ( +) AFR ( +) AMR ( +)	5.42 × 10^–4^	6.57 × 10^–5^	0.0763	ZBTB20
	chr5:166,060,445:G:A	rs79596863	2.75	9.94 × 10^–9^	EUR ( +) AFR ( +) AMR ( +)	0.348	3.68 × 10^–9^	0.0499	RPL7P20
	chr11:106,675,610:C:T	rs10890563	1.93	3.88 × 10^–8^	EUR ( +) AFR ( +) AMR ( +)	0.0103	1.13 × 10^–4^	0.00132	GUCY1A2
Pregnancy loss	chr15:93,045,079:C:G	rs62021480	3.53	4.50 × 10^–11^	EUR ( +) AFR ( +)	8.3 × 10^–7^	9.18 × 10^–6^	N/A	RGMA
	chr22:46,351,905:C:T	rs142795512	5.31	9.59 × 10^–10^	EUR ( +) AFR ( +)	0.5258	7.05 × 10^–10^	N/A	TRMU

Odds ratio (OR) is computed for the pregnancy loss, and GDM GWAS, while beta is computed for the gestational length (GWAS). “Effect Direction” reflects the direction of the odds ratio or beta of the SNP in each sub-cohort containing the variant (EUR, AFR, AMR; + indicates positive association,—indicates negative association).

Further investigation of the variants using annotation tools pinpoints potential functional effects. RegulomeDB scores all the associated variants as “regulatory” (scoring < 5 on the RegulomeDB scale). rs143149726, a downstream gene variant of *TRMU,* (TRNA 5-Methylaminomethyl-2-Thiouridylate Methyltransferase), scored the lowest score across all associated variants (indicating the greatest amount of evidence for the variant to be in a functional region). The variant had a RegulomeDB rank of 1f. with a probability of 1 (Table S1), indicating that it was “likely to affect binding and linked to expression of a gene target” given evidence of an expression quantitative trait locus (eQTL) and transcription factor binding or DNase peak. rs62021480, the top SNP association at the *RGMA* locus, encodes a 3 prime UTR variant for the gene, which is a glycoprotein that guides developing axons and may act as a tumor suppressor.

Through TWAS, we found a correlation between pregnancy loss and gene expression levels of *TTC38* (Tetratricopeptide Repeat Domain 38) in uterine (*Z* = 5.17, *P* = 2.39 × 10^–7^), liver (*Z* = 4.31, *P* = 1.62 × 10^–5^), and ovarian tissue (*Z* = 4.61, *P* = 3.91 × 10^–6^; Supplementary Figure S20, Supplementary Table S3). We were able to replicate previously reported associations (with *P* < 0.05) in three SNPs (out of 32; Table 3 and Supplementary Table S4), all near the *LINC01717* (Long Intergenic Non-Protein Coding RNA 1717) locus.

**Table 3:** Previously identified variants that were replicated in this study (association with relevant APO *P* < 0.05).

APO	rsID	OR (se)	P-value	Direction	Sample size	Mapped gene
Gestational length (EFO_0003917)	rs7093347	0.17 (0.05)	0.001749	+ + +	4646	ADAMTS14
	rs2963463	− 0.13 (0.05)	0.005615	–-	4646	EBF1, LINC02227
	rs113018921	0.24 (0.11)	0.025633	+ + -	4646	MSI2
	rs55889542	0.19 (0.09)	0.028307	+ + +	4646	KAZN
Preeclampsia (EFO_0000668)	rs4655789	1.36 (0.15)	0.012197	+—+	7480	WLS
	rs9663238	0.74 (0.06)	0.001554	–-	7366	HKDC1
GDM (EFO_0004593)	rs36090025	1.30 (0.11)	0.005257	+ + +	7366	TCF7L2
	rs10830962	1.28 (0.10)	0.005672	+—+	7366	MTNR1B, SNRPGP16
	rs9275373	1.34 (0.15)	0.0163	+ + +	7366	HLA-DQB1, MTCO3P1
Pregnancy loss (EFO_0006923)	rs12406463	1.58 (0.22)	0.005194	+ + ?	6855	LINC01717

The odds ratio (OR; standard error in parentheses), significance (*P*-value), and direction of the effect are shown for the meta-analysis of three sub-populations, as well as the closest gene(s) to the relevant marker.

### Gestational length

One multi-ancestry and one European-specific variant were implicated in the quantitative meta-analysis of gestational length (Supplementary Figures S13-S14). rs58548906 is an intergenic variant located on the X chromosome near *AC005052.1* and appears to be associated with a reduced gestational length (beta = − 1.43, CI = [− 1.94, − 0.921]) across African, Admixed American, and European ancestries. rs2550487, located on the 3 prime UTR of *WFDC1*, was found in the European sub-cohort only, and individuals carrying the minor allele are associated with lower gestational length (beta = − 1.28, CI = [0.215, − 1.70] ). Both SNPs show “minimal binding evidence” in their RegulomeDB ranking, with evidence of transcription factor binding or a DNase peak.

The genomic inflations of each sub-cohort GWAS ranged between λ = 0.99 and λ = 1.02, while the GWA meta-analysis across sub-cohorts did not show genomic inflation (Supplementary Figure S6). SNP-based heritability for this trait was *h*^2^ = 0.23 (SE = 0.13). We found evidence of association (*P* < 0.05) in 6 out of 79 SNPs previously reported to be associated with gestational length or similar phenotypes (Table 3, Table S4). These markers were clustered around four genes: *ADAMST14* (ADAM Metallopeptidase with Thrombospondin Type 1 Motif 14), *EBF1* (EBF transcription factor 1), *MSI2* (Musashi RNA Binding Protein 2), and *KAZN* (Kazrin, Periplakin Interacting Protein).

### Gestational diabetes mellitus

**Figure 2:**
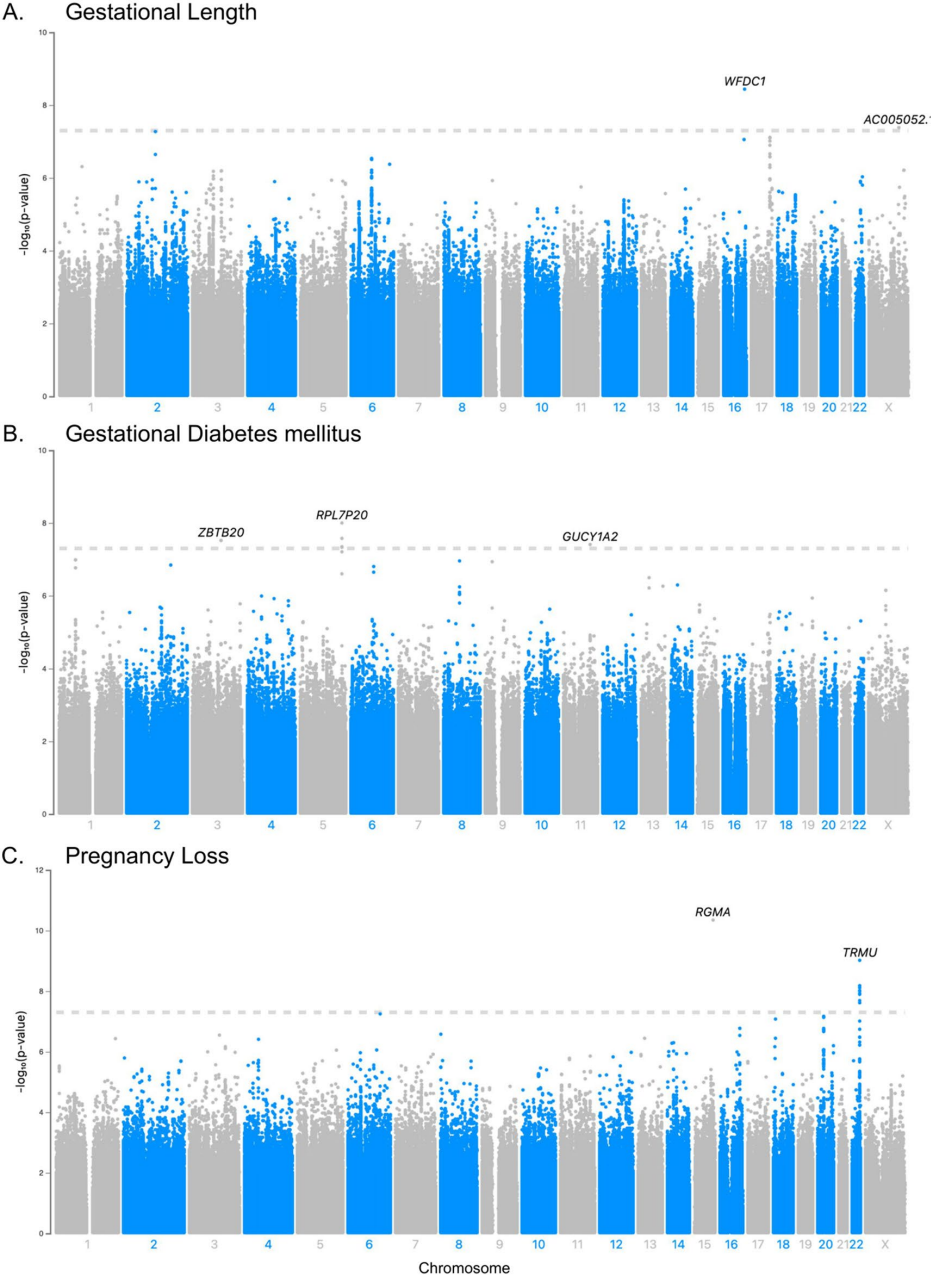
Manhattan Plot of GWAS Results: GWA meta-analysis was carried out using the inverse variance–weighted method implemented in GWAMA. The dotted line indicates the threshold for genome-wide significance (*P* < 5 × 10^–8^). (**A**) Gestational length; (**B**) GDM; (**C**) Pregnancy Loss.

Three significant loci were identified in the GWAS meta-analysis for GDM (Table 2, Table S2, Supplementary Figures S15-S19). A set of four SNPs appear in the intergenic region near *RPL7P20*, Ribosomal Protein L7 Pseudogene 20. The most significantly associated SNP in this set is the intergenic variant, rs79596863 (Table 2). The second significant locus includes intronic variants rs61167087 and rs72956265 in *ZBTB20*, a Zinc Finger and BTB Domain Containing 20. Lastly, rs10890563, a 3’UTR variant on the gene *GUCY1A2*, Guanylate Cyclase 1 Soluble Subunit Alpha 2, had a significant association with GDM as well. Several loci show suggestive associations in the meta-analysis, which we report in the supplementary text (Fig. 2; Table S2). We did not find genomic inflation in any of the ancestry groups or in the meta-analysis (λ = 1.001, Supplementary Figure S7), and we estimated SNP-based heritability to be *h*^2^ = 0.13 (SE = 0.085).

We also found a positive association with gestational diabetes for four previously reported variants (*P* < 0.05; Table 3). These SNPs were mapped near four genes: Hexokinase *HKDC1*, transcription factor *TCF7L2*, melatonin receptor *MTNR1B*, and major histocompatibility complex *HLADQB1*.

### Preeclampsia

We found no SNPs associated with preeclampsia (Supplementary Figure S8). Again, genomic inflation was low (λ = 1.005, Supplementary Figure S9), and SNP-based estimate of heritability was *h*^2^ = 0.02 (SE = 0.076). We confirmed 1 of 30 previously linked variants: rs4655789 (near gene *WLS*; Table S2).

## Discussion

We present a set of multi-ancestry GWAS of four of the most ubiquitous APOs. Overall, we identified variants at six novel loci associated with our APOs of interest, while also replicating 14 signals from previous studies. Results from the European-ancestry analyses also reveal two secondary signals at the *TRMU* locus, and the discovery of a new locus association with gestational length on *WFDC1*. Using a Bayesian fine-mapping approach, we further narrowed the list of putative causal variants to those within a credible posterior probability of at least 95%. The candidate genetic markers identified in the present study provide a starting point for molecular exploration of each APO of interest.

The cluster of 11 significant variants at the *TRMU* locus in the pregnancy loss GWAS are characterized as having a significant regulatory role in binding and gene expression given high-throughput experimental data from RegulomeDB. Indeed, the expression quantitative trait locus (eQTL) annotation from RegulomeDB and VEP indicate that the SNPs in the aforementioned cluster (Table S1) mediate gene expression across several two genes located on chromosome 22q13.31: *TRMU* and *CELSR1*. Additionally, TWAS analysis implicates the gene *TTC38* in pregnancy loss across three tissue types: liver, ovary, and uterus. A top SNP in this locus, rs114058457 (Table S1), was previously found by Jansen et al. as an eQTL for *TTC38* in blood in a cohort of 4,896 subjects of European ancestry (FDR-corrected *P* < 1.34 × 10^–5^)^32^. Overall, there appears to be consistent evidence mapping the top SNPs from the chromosome 22q13.31 locus to biologically meaningful roles.

While the preliminary annotations of the chromosome 22q13.3 SNP cluster seem to be promising, further work is necessary to pinpoint the mechanistic contribution of the four target genes. Previous work has shown that *TRMU* encodes a mitochondria-specific tRNA-modifying enzyme and plays a key part in mitochondrial translation^33^. Aberrant expression of *TRMU* is likely pathogenic to humans early in life, as variants in *TRMU* have been linked to several disease phenotypes, including infantile liver failure^33^, and infantile reversible cytochrome c oxidase deficiency^34^. Next, *TTC38*, implicated through the TWAS, has been posited as a factor associated with kidney development^35^, while *CELSR1* is highly prevalent in embryonic tissues^36,37^ and linchpins embryonic development across humans and other vertebrates^38,39^. The genes we have outlined here span a diverse set of functions, however we speculate that an underlying commonality is that they act as important contributors to early development.

Rs62021480, located near *RGMA* and associated with greater risk of pregnancy loss, plays a role in transcription factor binding and lies in an open chromatin region, however the evidence for a regulatory role is minimal. Still, rs62021480 lies within the 3’ UTR of *RGMA*, and therefore may have post-transcriptional regulatory function. *RGMA* is a repulsive guidance molecule for axons of the retina, which is a pivotal step in the developing brain^41^.

Interpretation of the results from the remaining two GWAS is less straightforward as the associated SNPs have greater ambiguity from a functional perspective. The top GDM SNPs identified localize to three genes: *RPL7P20*, *ZBTB20*, and *GUCY1A2*. While *RPL7P20* codes for a pseudogene, several studies have made associations between SNPs at this locus and increased heart rate^42^ as well as general cognition^43,44^. The zinc finger *ZBTB20* has been linked to Primrose syndrome^45^, which may include insulin-resistant diabetes and other metabolic disruptions. This transcription factor regulates beta-cell function in mice^46^ and has been hypothesized to be involved in the control of glucose metabolism. *GUCY1A2* (a guanylate cyclase, GTP-binding protein) has shown high expression levels in placenta and was differentially expressed in rat placentas responding to hyperglycemia^47^. In addition, *GUCY1A2* has been inferred to be associated with diabetes through exposure to several toxins (reported in the Comparative Toxicogenomics Database^48^).

Two significant SNPs map to two separate loci in the GWAS of gestational length: *AC005052.1* and *WFDC1*. While the gene profile of *AC005052.1* is relatively unknown, *WFDC1* modulates inflammatory response^49^. Inflammation has a clearly established link to preterm birth^50^ and dysregulation of the immune system through inflammation may lead to harmful effects on pregnancy^51^. These initial findings suggest potential mechanisms through which the SNP findings may shorten gestational length during pregnancy.

In conjunction with the GWAS findings, nuMoM2b offers a uniquely valuable resource for genetic studies on two levels. First, the dataset spans over 4,600 features per subject^12^, representing a multimodal array of clinical, biological, and environmental factors influencing pregnancy. The deep, longitudinal phenotyping of the cohort presents varied opportunities for additional GWAS and downstream analyses. Second, another advantage of this study and any future genetic studies performed using nuMoM2b’s multi-ancestry population is the potential for better genetic signal triangulation in the face of confounds. As nuMoM2b contains several distant ancestral populations, meta-analyses of GWAS from each ancestry group takes advantage of naturally occurring differences in linkage disequilibrium between SNPs to disentangle false positives from the most probable causal variant. In particular, having an African ancestry sub-cohort is not only inherently beneficial to study, but also helpful in that African populations contain a more diverse set of haplotypes^52^ that can hone in on causal variant signals from a more homogeneous population. The preprocessing pipeline established in this study can be used in future studies to study additional variants in an ancestry-stratified manner.

There are still important limitations. When considering many of the disease phenotypes in nuMoM2b, there is an imbalanced breakdown of subjects in terms of both case–control status and genetic ancestry. This imbalance persists in the analyses presented here—indeed, the pregnancy GWAS meta-analysis excluded the Admixed American sub-cohort due to extreme class imbalance, and the meta-analysis results contain > 70% of individuals of European ancestry, despite the inclusion of diverse ancestry populations. Our results would therefore benefit from further validation cohorts, particularly of individuals of non-European genetic ancestry, to improve with broad applicability of the findings. Future studies may want to study quantitative traits to improve statistical power given modest sample sizes.

This study confirmed previously reported associations between several SNPs and APOs but failed to do so for most markers queried. This result is not unexpected, however, since low levels of replication in GWAS are not uncommon^53,54^. While we investigated the functional role of our variants of interest through fine-mapping approaches and annotation tools, the APOs studied in this paper are ultimately defined by complex genetic architectures. The effect sizes observed for individual SNPs suggest that the APOs studied are highly polygenic. These findings imply that, rather than aiming for genetic screening at one or a few markers, better prediction of these APOs will come through the modeling of genetic signals genome-wide (e.g., through polygenic risk scoring) together with non-genetic factors. Examination of gene-by-environment interactions, which disentangle the nonlinear interplay of variants and external factors on phenotype, may further illuminate drivers of APOs^55^. Such efforts are already underway, with auspicious results for the prediction of GDM through the combination of polygenic risk scores and behavioral data^56^. It is also possible that these complex phenotypes are largely controlled by rare genetic variants^57^ for which GWAS are not adequate. Identifying rare causative variants would require sequencing efforts including whole-genome and whole-exome sequencing, as well as larger study populations of diverse genetic ancestry.

The results of our multi-ancestry study highlight the role of previously unknown loci across several APOs: gestational length, GDM, pregnancy loss, and preeclampsia. We unearth potential contributors to preterm birth at multiple levels of granularity—SNP, gene, and tissue. We also confirm previously identified genetic associations for preterm birth, GDM, and preeclampsia. These findings broaden our understanding of the complex polygenic nature of the APOs studied, informing further research directions and enabling downstream genetic analyses.

### Supplementary Information

Supplementary Information 1.

Supplementary Information 2.

Supplementary Information 3.

Supplementary Information 4.

Supplementary Information 5.

## Data Availability

Summary statistics of the GWA meta-analysis and corresponding genetic data will be available through dbGaP under study accession phs002808.v1.p1 (https://www.ncbi.nlm.nih.gov/projects/gap/cgi-bin/study.cgi?study_id=phs002808.v1.p1).
