# Supplementary material for: Genetic polymorphisms associated with adverse pregnancy outcomes in nulliparas
Source: Sci Rep. 2024 May 7;14:10514. doi: 10.1038/s41598-024-61218-9 (PMC11076516; doi:10.1038/s41598-024-61218-9)
Supplement: Supplementary file 1 — Supplementary Information 1. [file 41598_2024_61218_MOESM1_ESM.docx]

**Supplementary Figures for “Genetic Polymorphisms Associated with Adverse Pregnancy Outcomes in Nulliparas**”**:**


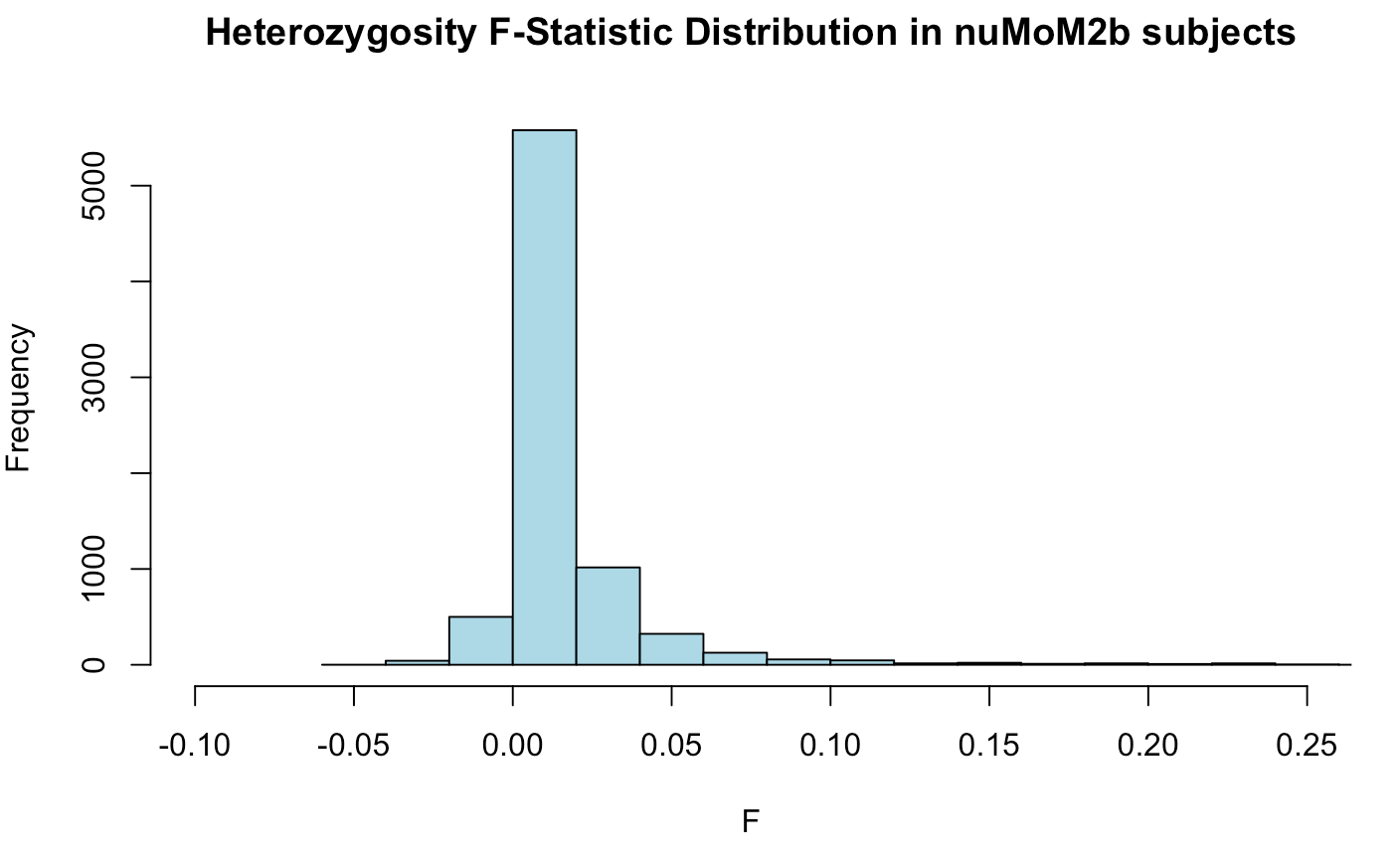


Figure S1: Inbreeding coefficient (F-statistic) distribution for nuMoM2b subjects. Individual heterozygosity level was estimated using PLINK1.9.


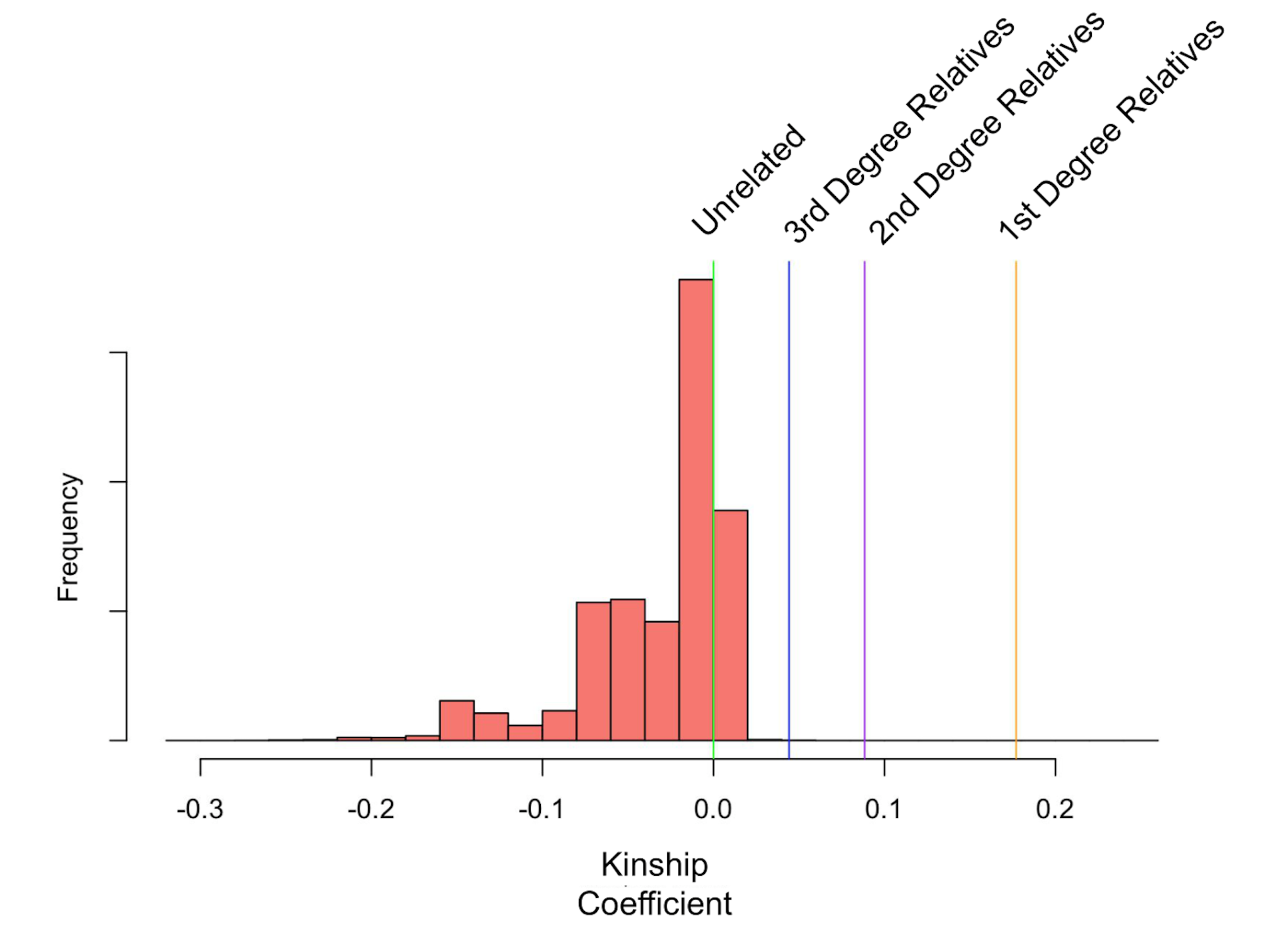


Figure S2: Estimated pairwise kinship coefficients in nuMoM2b subjects using the KING-Robust algorithm. Close relatives can be inferred based on the following cutoffs (shown by the vertical lines): >0.354 = duplicate/monozygotic twin, [0.177, 0.354] = 1st-degree relationships, [0.0884, 0.177] = 2nd-degree relationships, [0.0442, 0.0884] = 3rd-degree relationships.

**
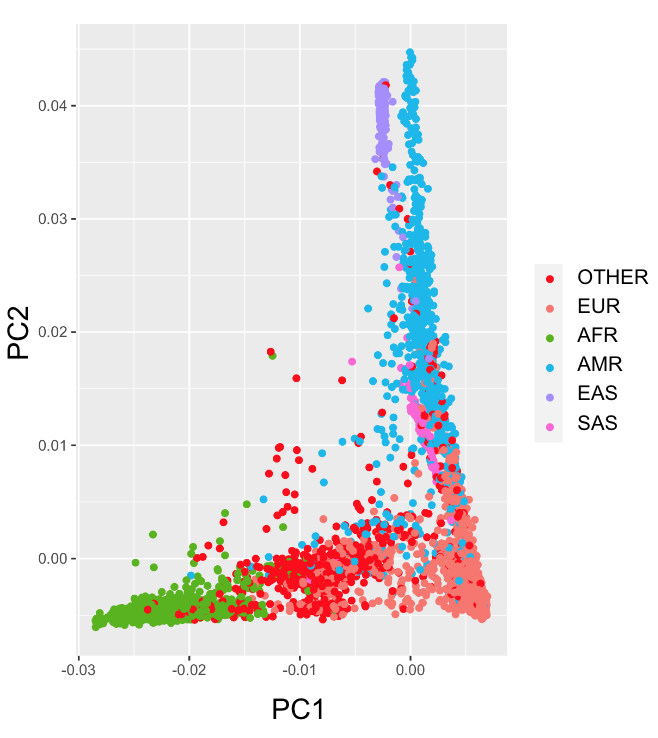
**

Figure S3: Ancestry analysis of the nuMoM2b study participants. The first two principal components computed by PLINK1.9 are color coded according to the participant’s dominant ancestry as identified by SNPweights markers (>50%): EUR - European, AFR - African, AMR - Admixed Americans, EAS - East Asian, SAS - South Asian. All subjects identified as “OTHER” do not have any ancestry percentage that is greater than 50%.


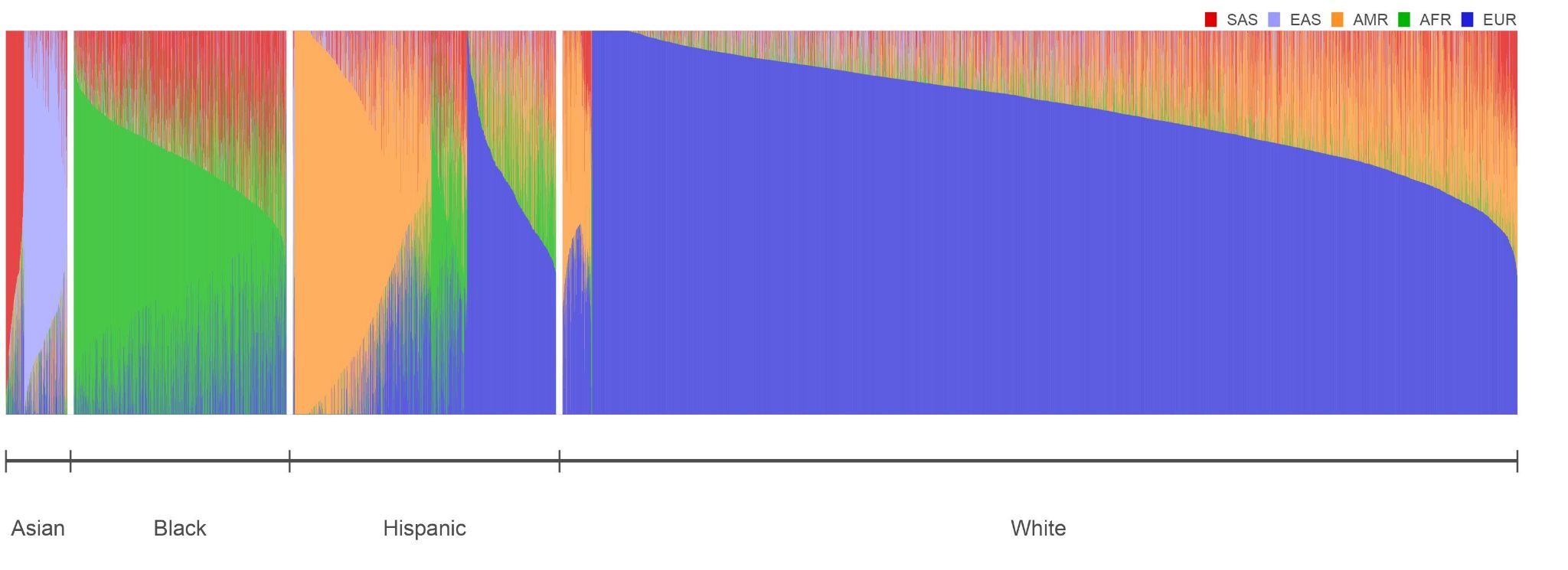


Figure S4: Ancestry proportions of all subjects, who are grouped by their self-reported race, in the nuMoM2b cohort, as determined by SNPWeights v.2.1. Each color represents a different ancestral component, using the 1000 Genomes super populations: South-Asians (SAS), East-Asians (EAS), Americans (AMR), Africans (AFR), Europeans (EUR).


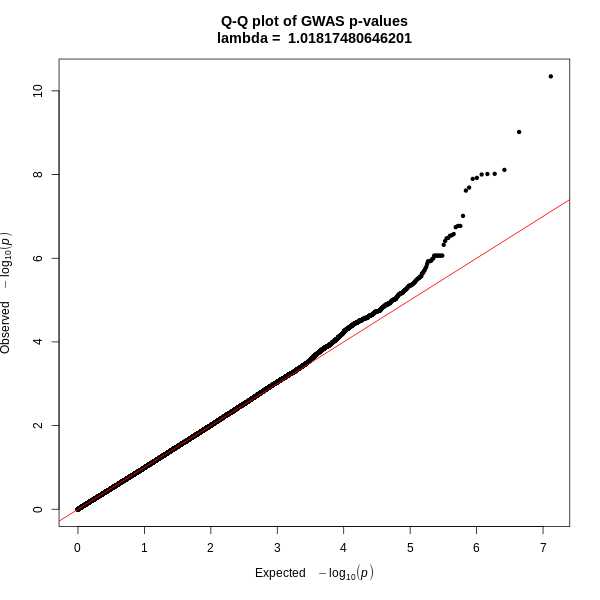


Figure S5: Q-Q Plot Pregnancy Loss Meta-Analysis

**
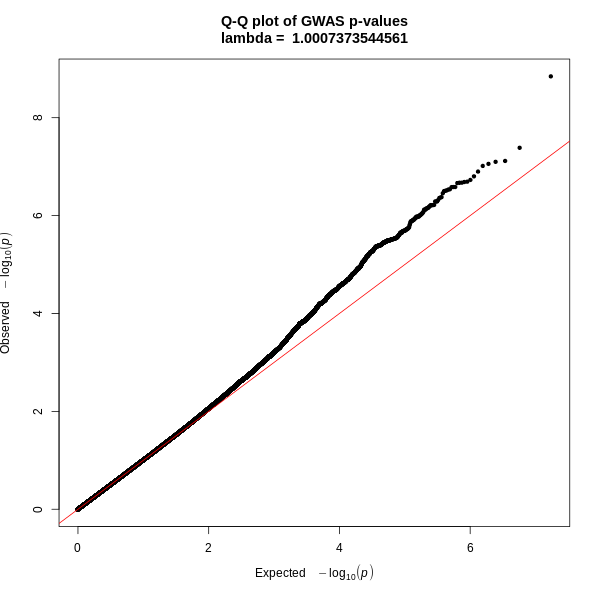
**

Figure S6: Q-Q Plot Gestation Length Meta-Analysis


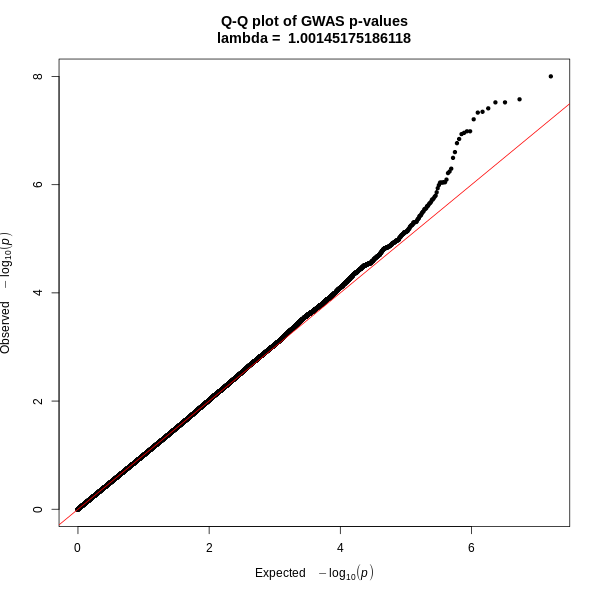


Figure S7: Q-Q Plot Gestational Diabetes Meta-Analysis


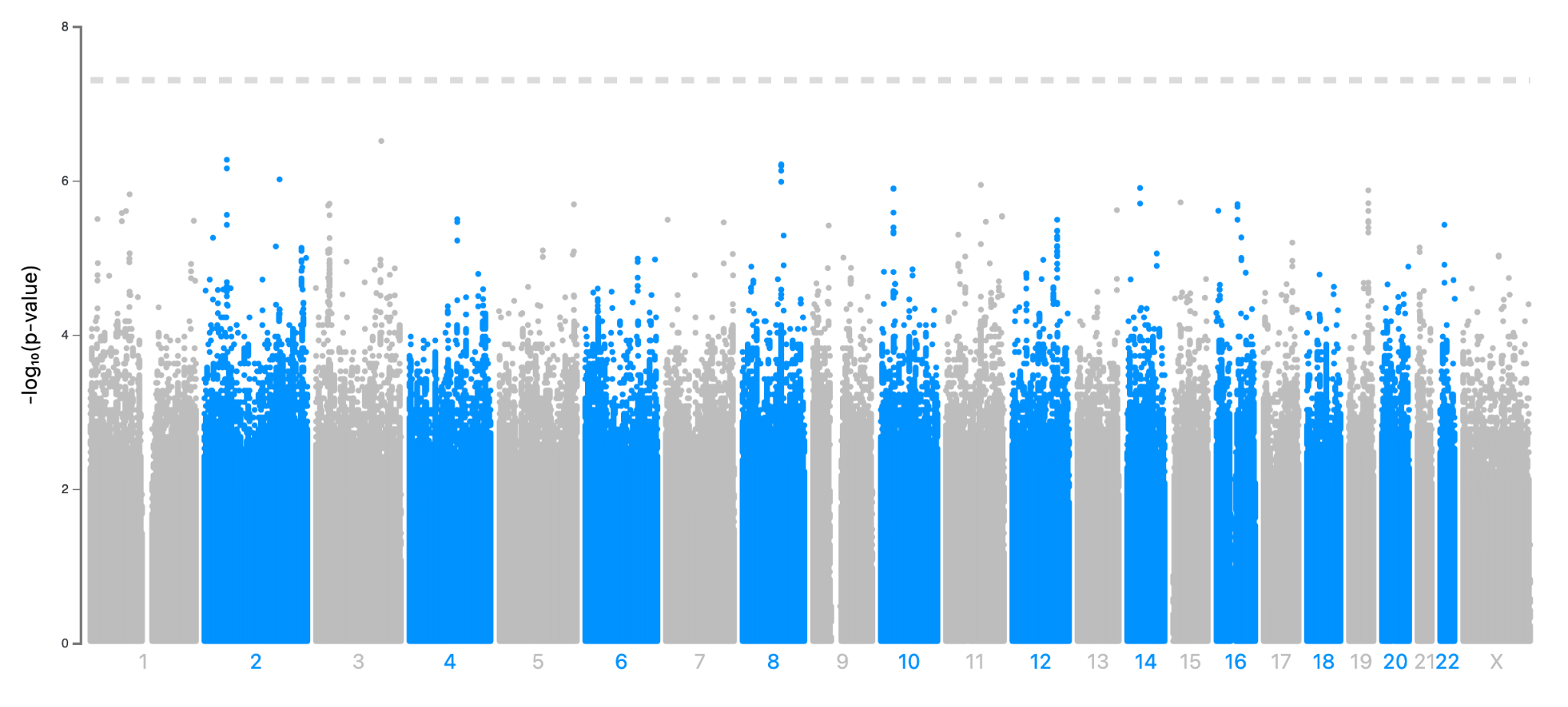


Figure S8: Manhattan Plot of the Meta-Analysis of Preeclampsia.


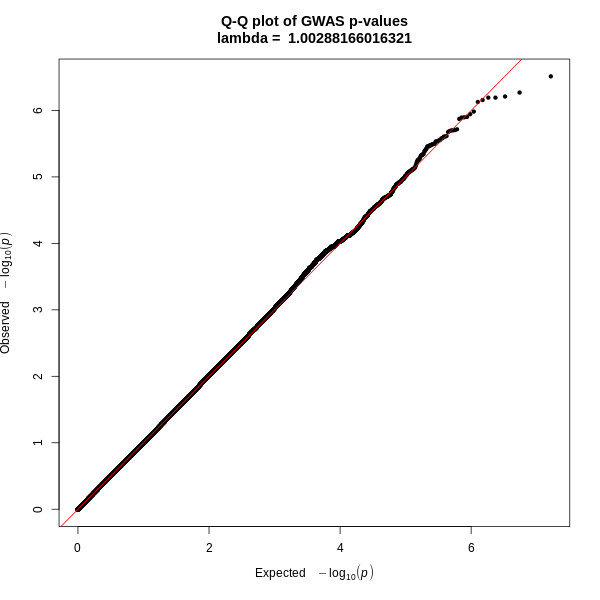


Figure S9: Q-Q Plot Preeclampsia Meta-Analysis


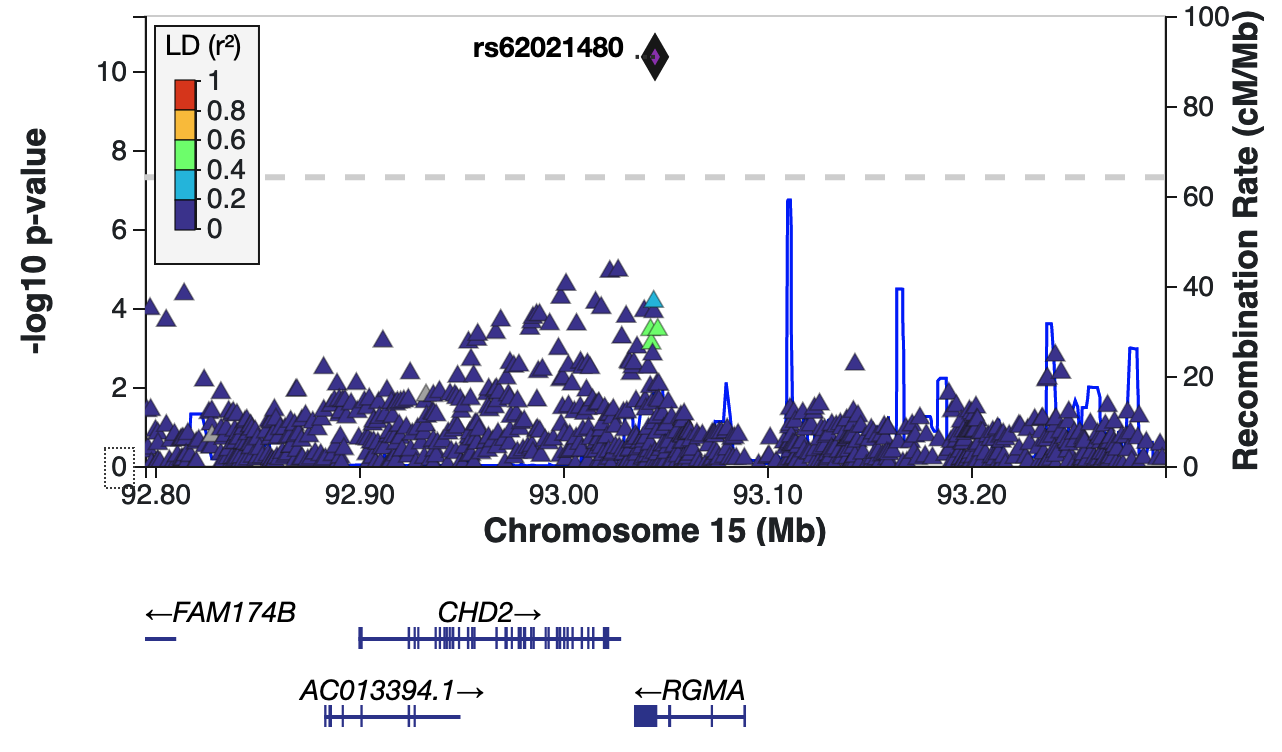


Figure S10: Linkage disequilibrium plot of top SNP rs62021480 in the pregnancy loss GWAS


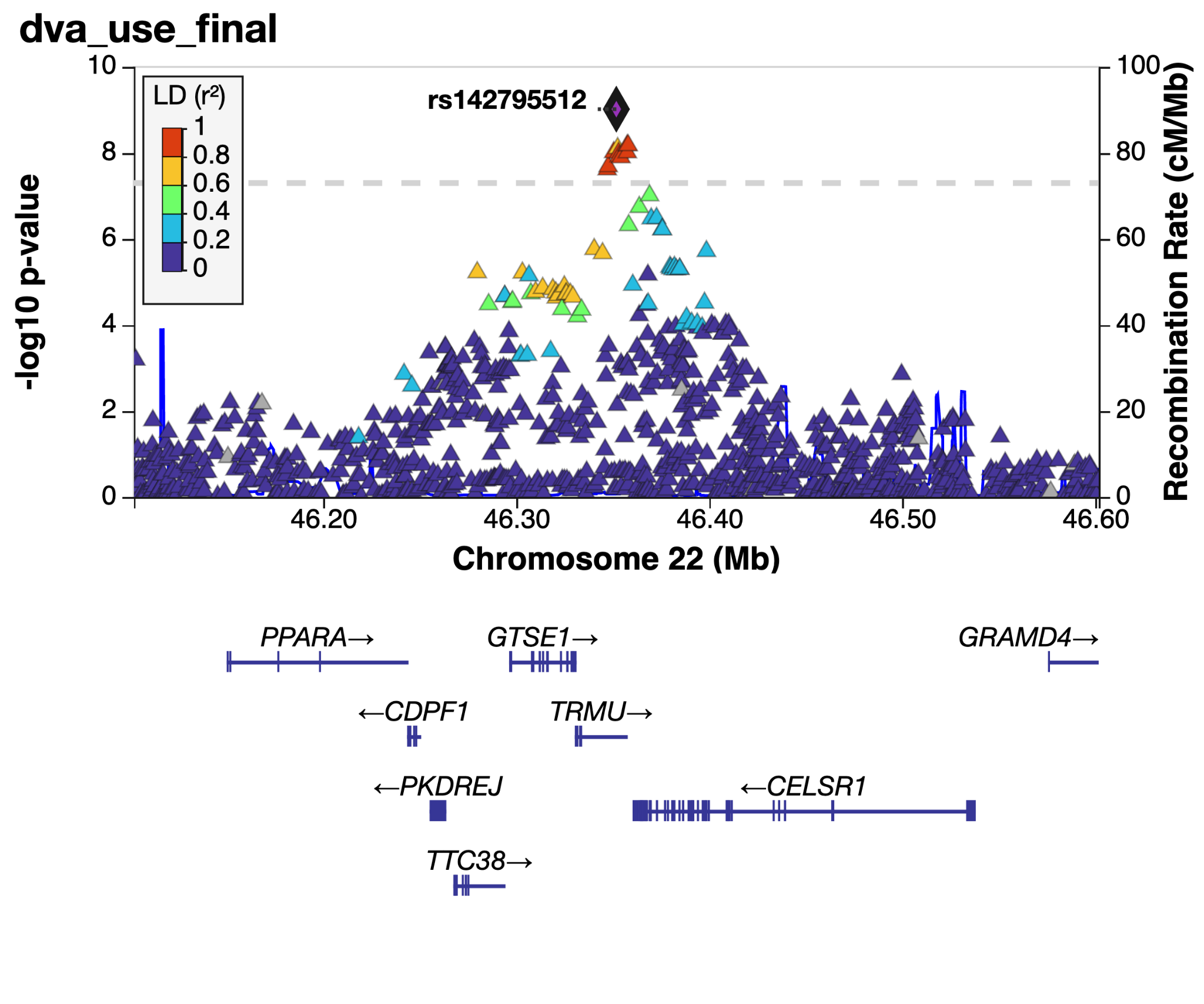


Figure S11: Linkage disequilibrium plot of top SNP rs142795512 from the pregnancy loss GWAS


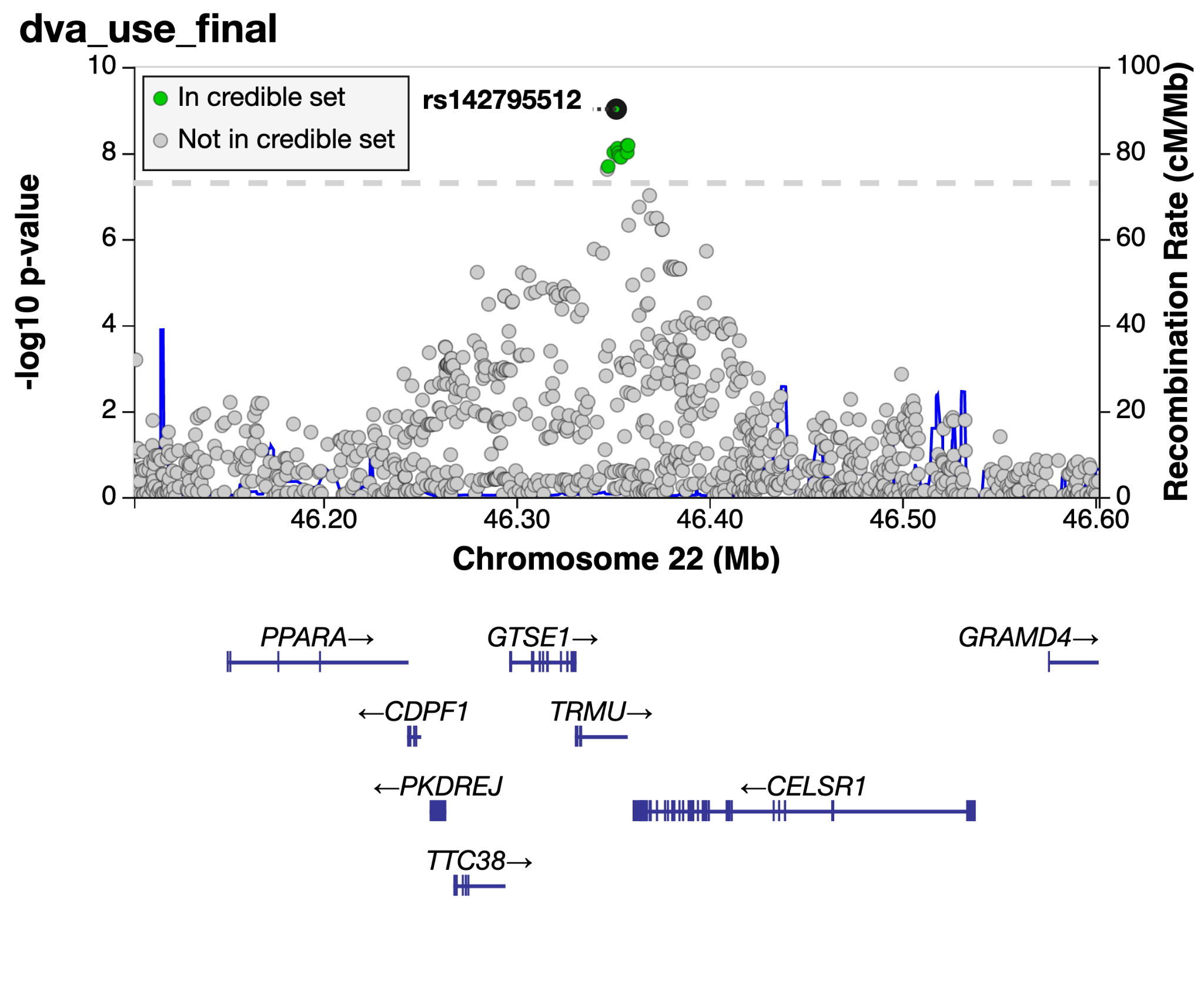


Figure S12: The 95% credible set of causal SNPs at the *TRMU* locus from the pregnancy loss GWAS


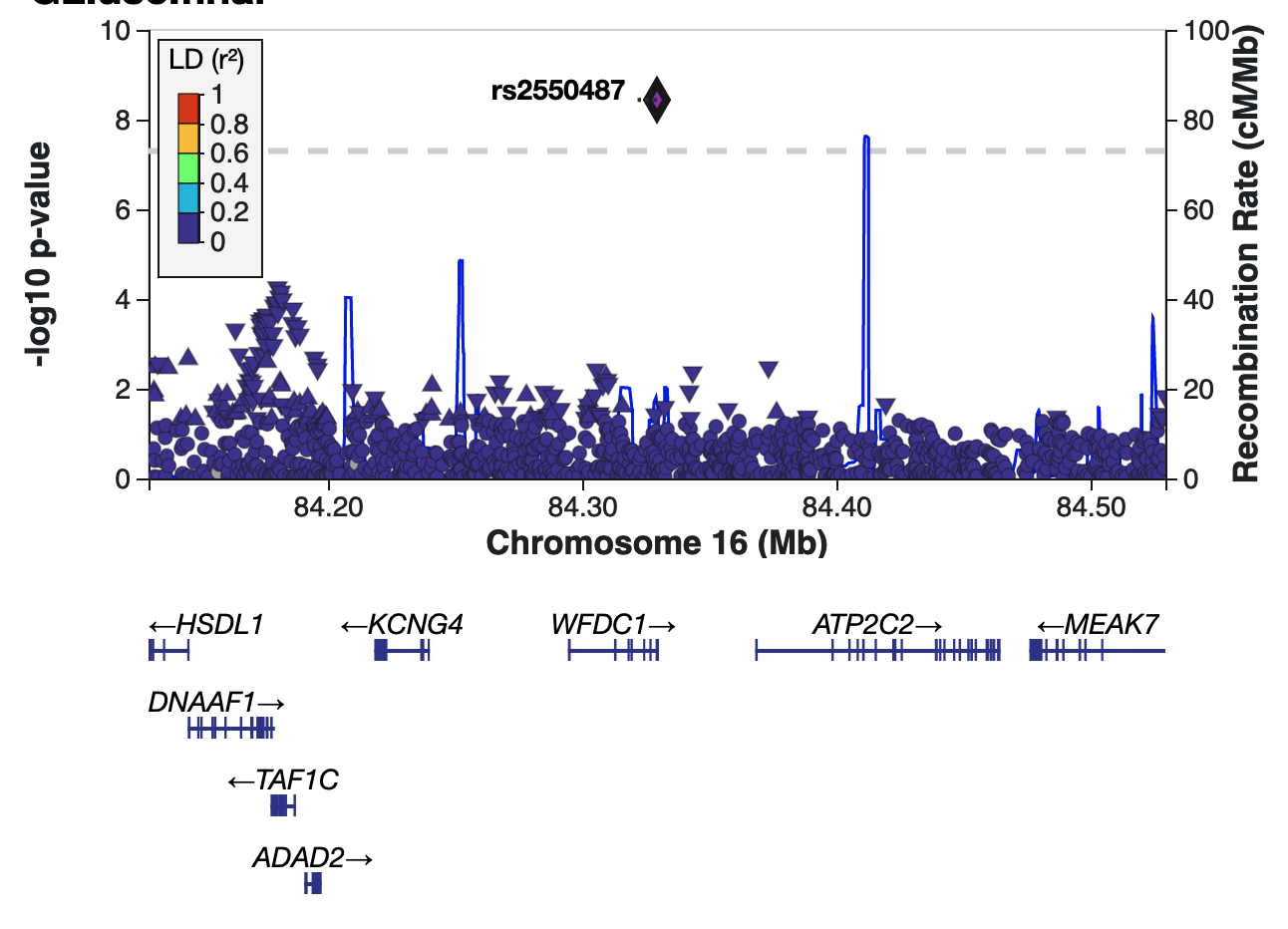


Figure S13: Linkage disequilibrium plot of top SNP rs2550487 from the gestational length GWAS


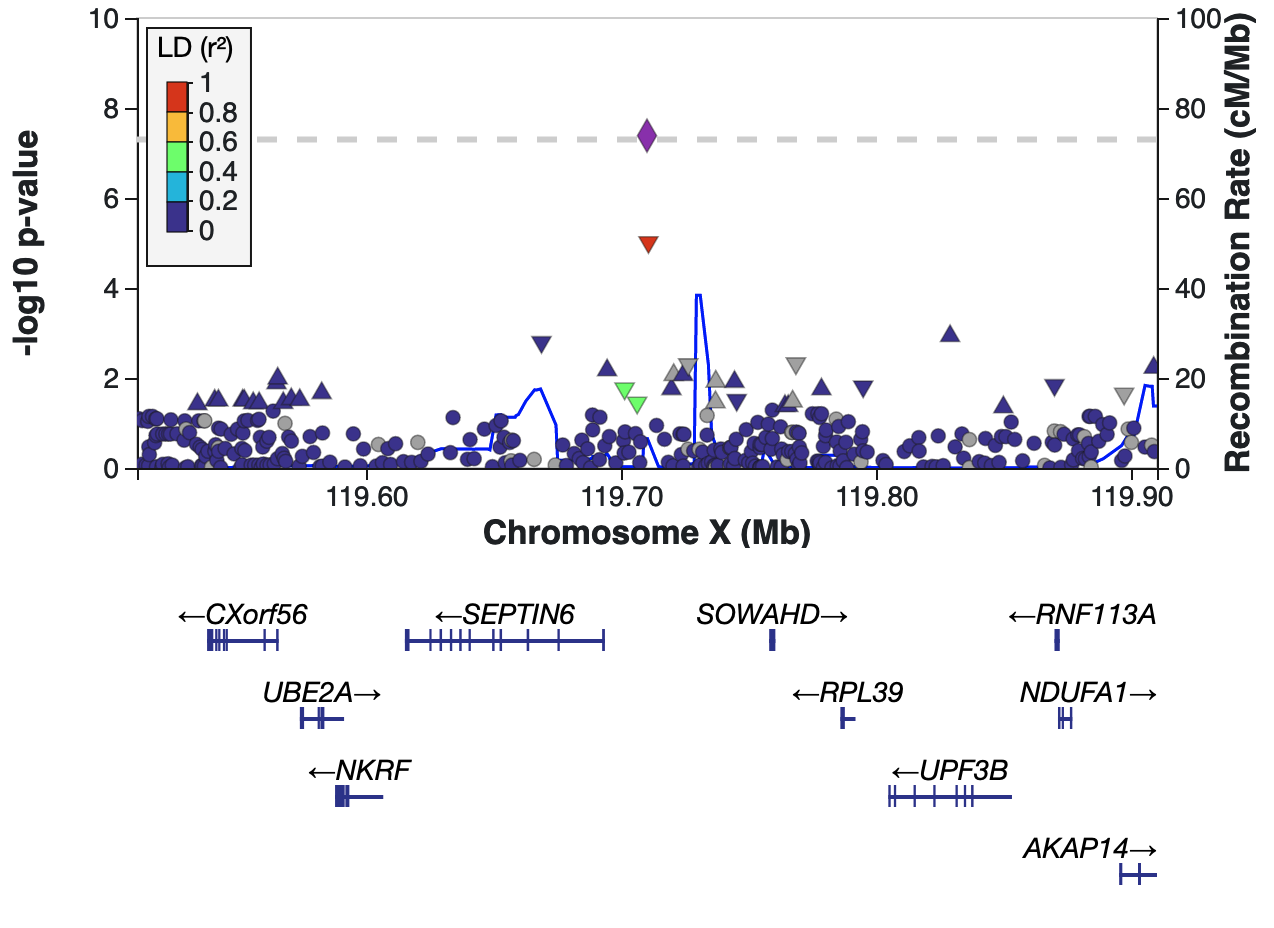


Figure S14: Linkage disequilibrium plot of top SNP rs2550487 from the gestational length GWAS


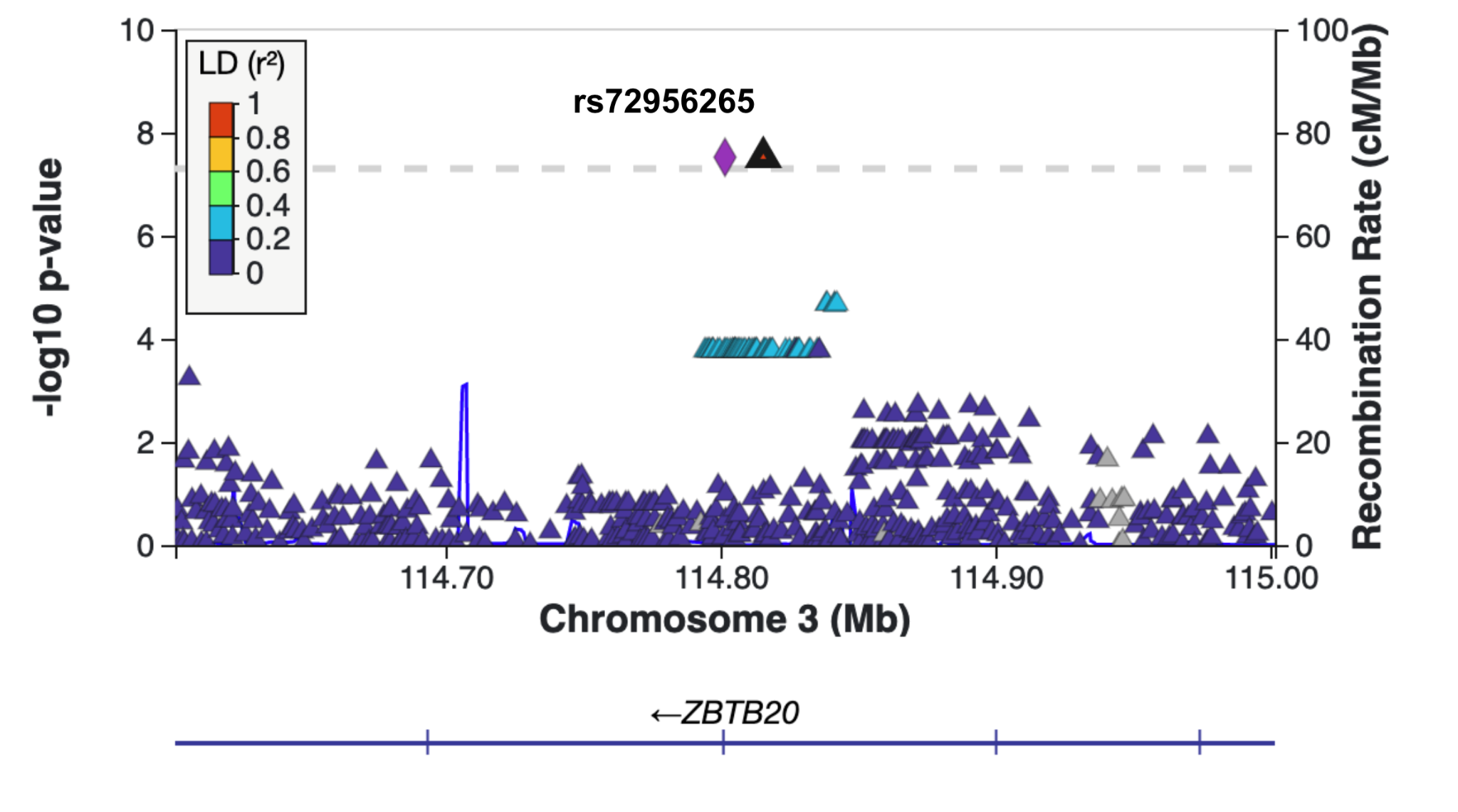


Figure S15: Linkage disequilibrium plot of top SNP rs72956265 from the GDM GWAS


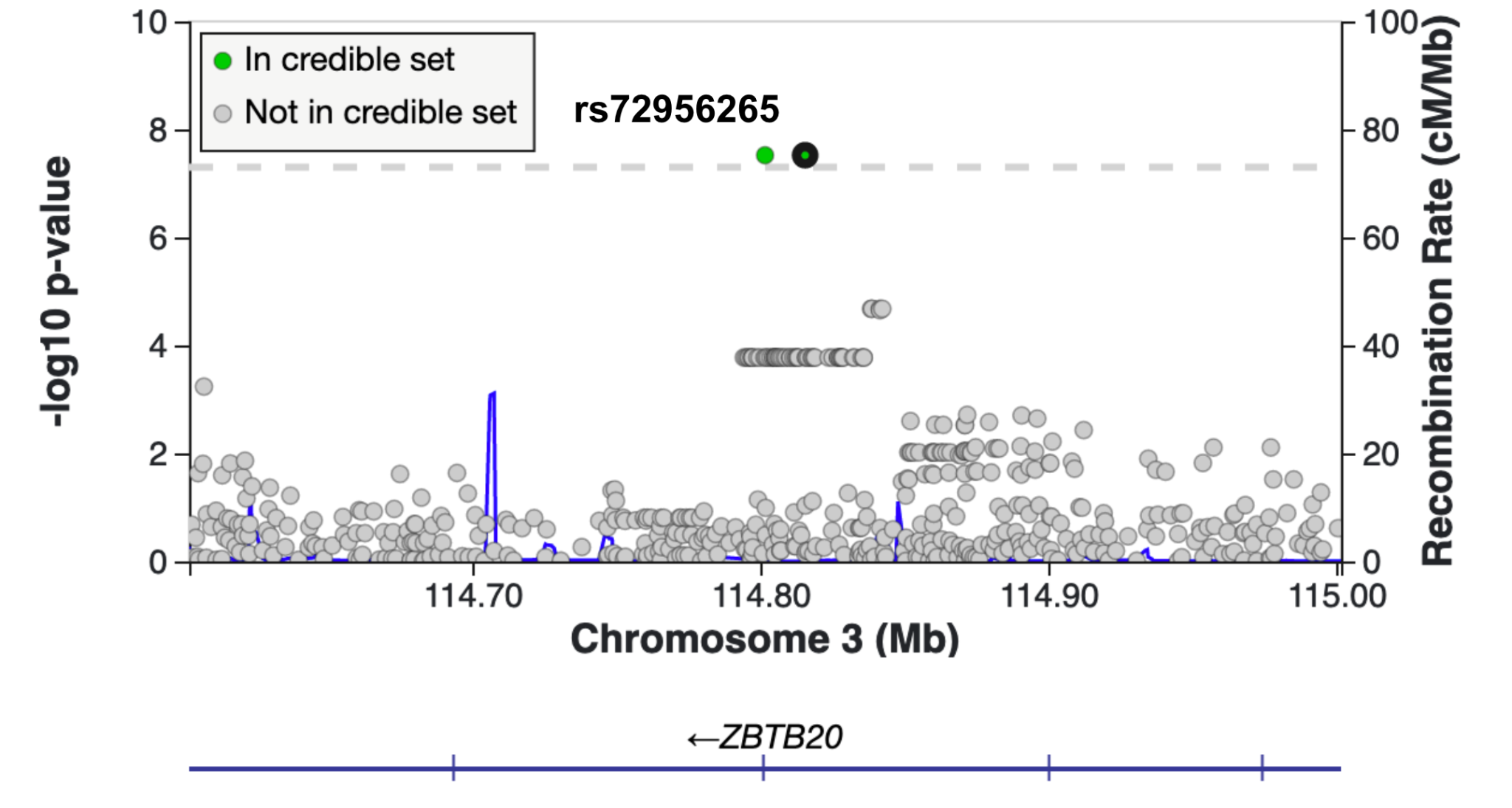


Figure S16: The 95% credible set of causal SNPs at the *ZBTB20* locus from the GDM GWAS


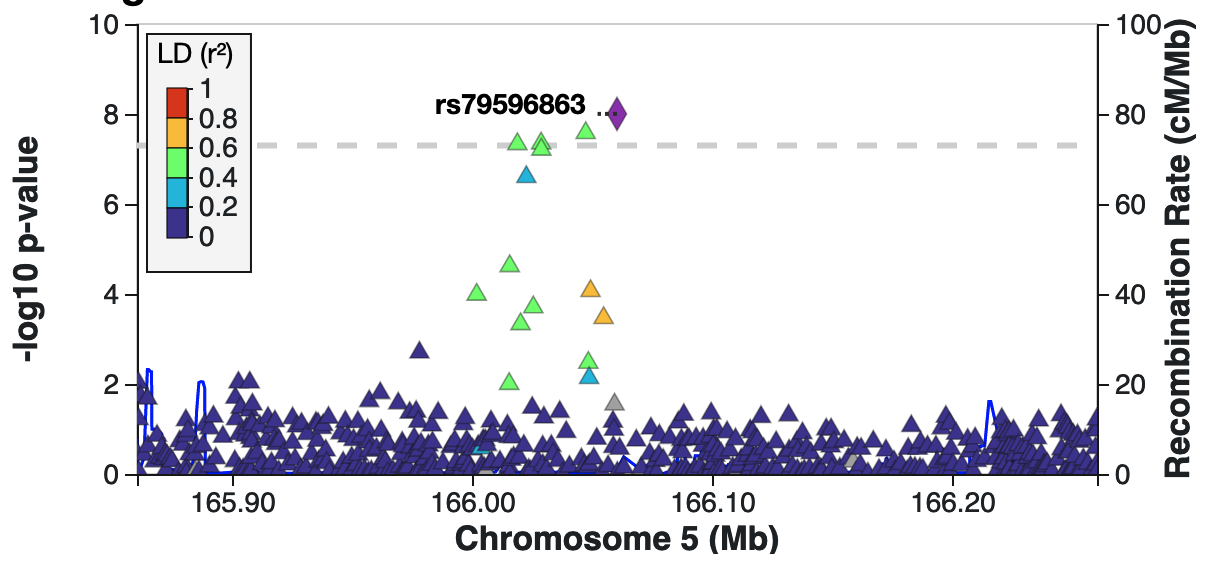


Figure S17: Linkage disequilibrium plot of top SNP rs79596863 from the GDM GWAS


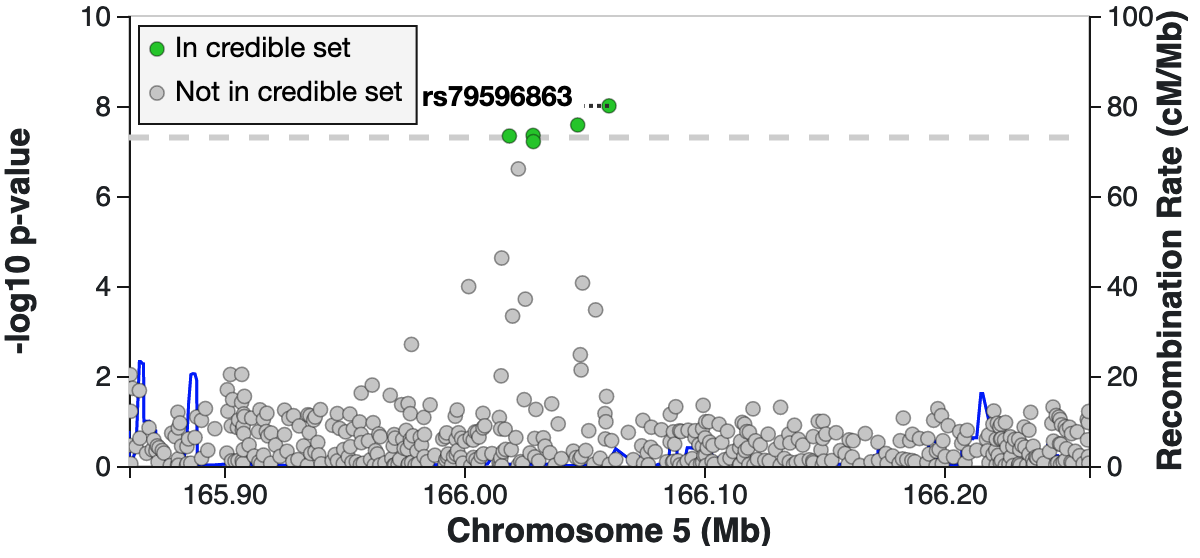


Figure S18: The 95% credible set of causal SNPs at the *RPL7P20* locus from the GDM GWAS


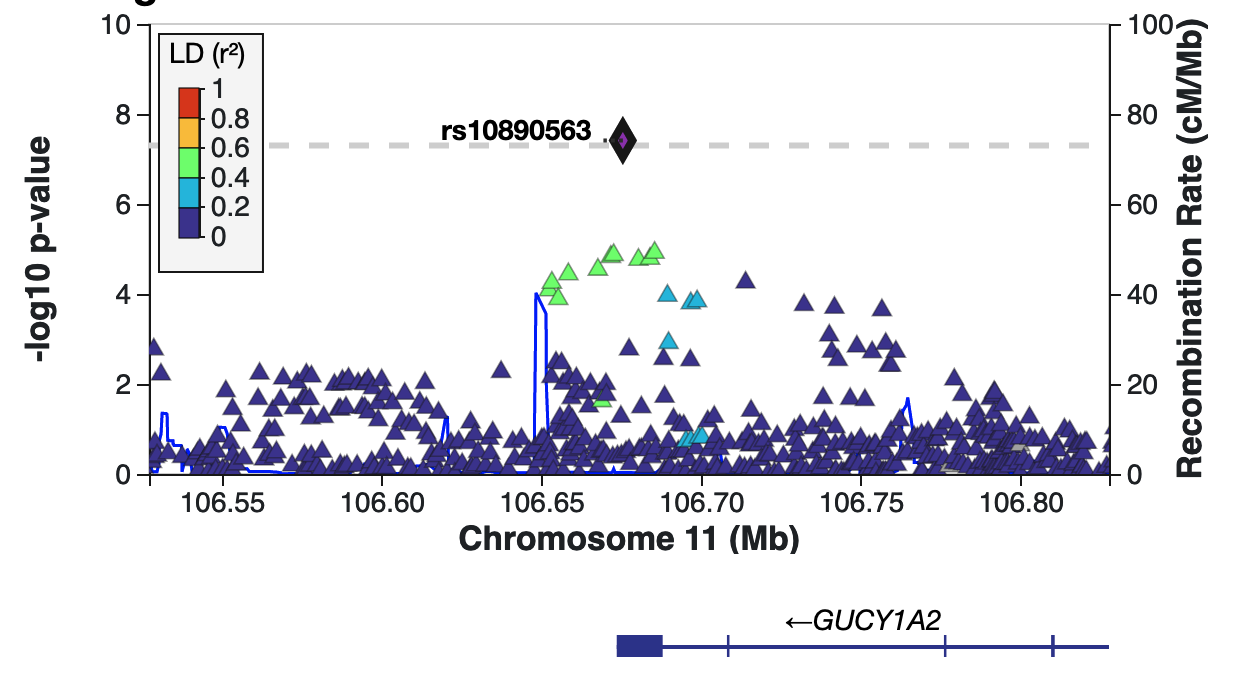


Figure S19: Linkage disequilibrium plot of top SNP rs10890563 from the GDM GWAS


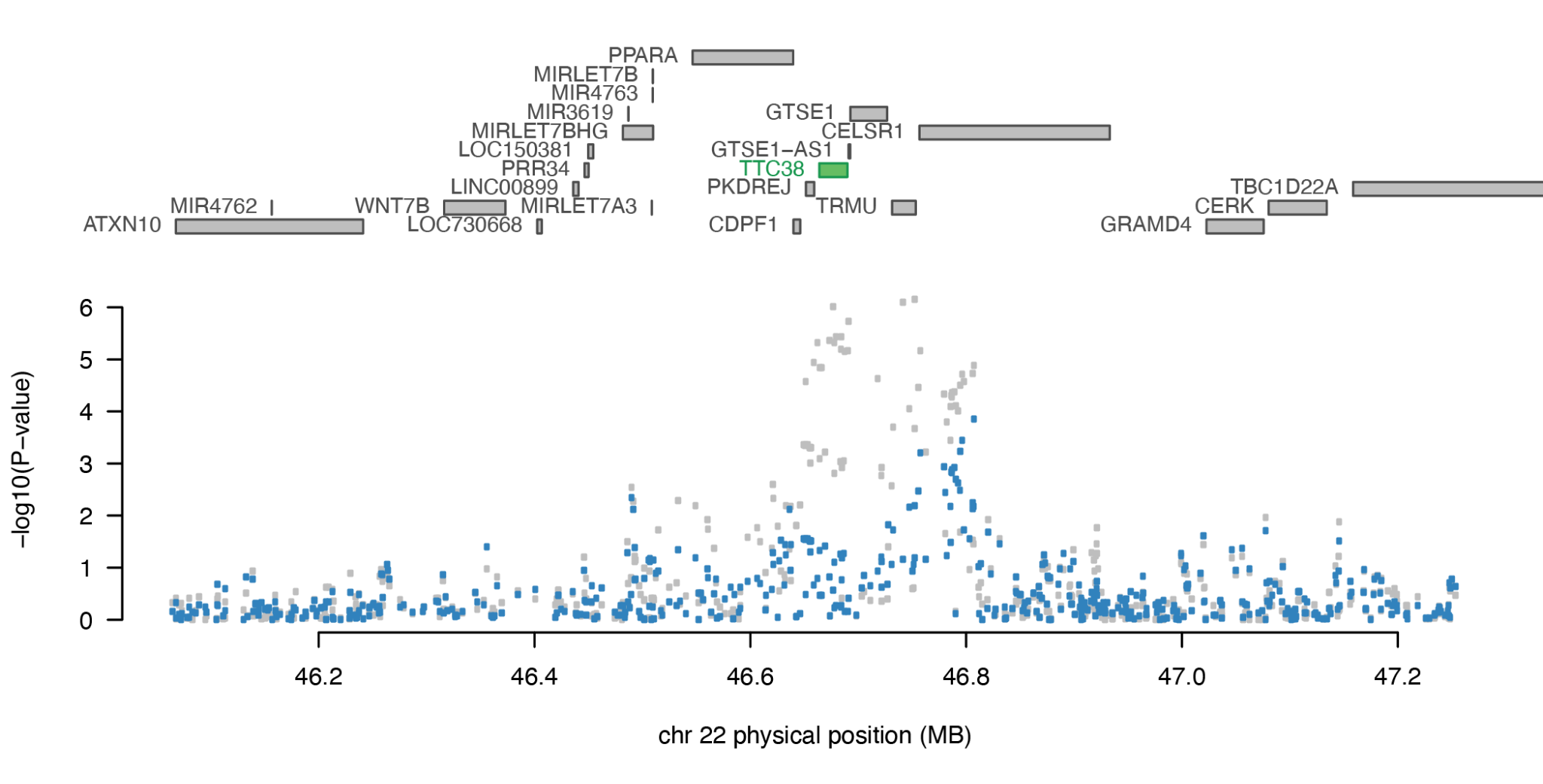


Figure S20. Genomic region that showed transcriptome-wide significant association with pregnancy loss. (eQTL rs7290732; eQTL Z-score = 5.11, ‘eNet’ model, TWAS Z-score =5.16, TWAS *P*-value = 2.39e-7). Top: Genes in the region. In green, the gene with significant association. Bottom: GWAS results for the region before (grey) and after (blue) conditioning on TTC38.
